# The impact of enterprise resilience and HRM practices on performance: Findings from fsQCA

**DOI:** 10.3389/fpsyg.2023.1114087

**Published:** 2023-02-13

**Authors:** Bo Liu, Yan Li, LinHua Tang, Yipeng Zhao, Haodong Chang

**Affiliations:** ^1^College of Management Science, Chengdu University of Technology, Chengdu, China; ^2^College of Mathematics and Physics, Chengdu University of Technology, Chengdu, China

**Keywords:** enterprise resilience, human resource management practice, enterprise performance, combination configuration, fuzzy set qualitative comparative analysis

## Abstract

**Background:**

Both enterprise resilience and HRM practices can have a positive impact on enterprise performance. The impact of enterprise resilience or human resource management (HRM) practices on enterprise performance independently has been studied widely. But few studies have combined the above two aspects to discuss their impact on enterprise performance.

**Objective:**

In order to provide positive conclusions for improving enterprise performance, the theoretical model is established to expound the relationship between enterprise resilience, HRM practices including their internal influencing factors and enterprise performance. According to this model, a series of hypotheses about the influence of the combination from these internal factors on enterprise performance are presented.

**Method:**

Based on the statistical data of the questionnaire survey with managers and general employees at different levels in enterprises as respondents, the correctness of these hypotheses is proved by the fuzzy set qualitative comparative analysis (fsQCA) method.

**Results and discussion:**

The impact of the combination of enterprise resilience for high enterprise performance is shown in Table 3. The positive impact on the configuration of HRM practices for enterprise performance is shown in Table 4. The influences of the various combinations of internal factors about enterprise resilience and HRM practices on enterprise performance are shown in Table 5. From Table 4, it is discovered that performance appraisal and training have a significant positive effect on high enterprise performance. From Table 5, it is found that information sharing capabilities play a critical role, and enterprise resilience capabilities have a relatively positive impact on enterprise performance. Therefore, managers need to seek the development of enterprise resilience and HRM practices simultaneously and choose the most suitable combination configuration according to the actual situation of the enterprise itself. Moreover, a meeting system should be set up to ensure the transmission of internal information efficiently and accurately.

## 1. Introduction

Due to the intensifying competition and the various uncertainties, the rapidly changes of both the external market environment and the internal organization put forward the higher requirements for enterprise management. Hamel and Valikangas (2003) pointed out that successful organizations need the ability to dynamically reshape their business models and strategies in response to environmental changes, i.e., resilience. Sutcliffe and Vogus (2003) stated that a resilient enterprise is able to proactively respond and adjust to external changes. Ortiz-de-Mandojana and Bansal (2015) argued that a resilient company usually has a higher survival rate and longer-term profitability than an inelastic company. Therefore, in order to quickly respond to the ever-changing changes, it is particularly important to study the elasticity of enterprises to adjust the internal organization and process in time. Currently, most research on enterprise resilience has focused on conceptual models of resilience (Hillmann and Guenther, 2020), and there are very few quantitative studies. Moreover, even when quantitative research is conducted, it measures the resilience level of companies only by designing contingency measures for several types of emergencies, and a deep and comprehensive understanding of enterprise resilience is lacking (Sanchis and Poler, 2019).

In addition, current researches mainly focus on the impact of one of the two aspects, enterprise resilience and human resource management (HRM) practices in firm performance, and there is a lack of research on the effect of the combination of the two on enterprise performance (Katou and Budhwar, 2010). At present, some studies show that HRM practices can impact enterprise performance. However, it mainly focuses on the six internal influencing factors of HRM practices in public service enterprises, which respectively have a positive impact on enterprise performance (Della Torre, 2019). The above study lacks the discussion about the effect of the combination of the six internal influencing factors on performance. In fact, the various combinations of internal factors about HRM practices play a more prominent role in enterprise performance than a single factor (Ichniowski and Shaw, 1999).

Therefore, in this paper a theoretical model is established to expound the relationship of the combination of the two on enterprise performance firstly. According to this model, a series of hypotheses about the influence of the combination from these internal factors on enterprise performance are presented. Then, based on the statistical data of the questionnaire survey, the correctness of these hypotheses is proved by the fuzzy set qualitative comparative analysis method (fsQCA). At the same time, the analysis identifies various alternative paths for achieving high or non-high enterprise performance.

## 2. Literature review

Resilience, a physical term, refers to the ability of an object to return to its original state after deformation. However, the concept of enterprise resilience has not reached a consensus in the field of management. Burnard and Bhamra (2011) consider the resilience within an organization to be a potential framework fitting for organizational development, which can overcome disruption and inconsistency. According to Annarelli and Nonino (2016), it is a necessary strategic awareness to prepare for disruptive and unforeseen events in advance for enterprise resilience. Kamalahmadi and Parast (2016) think enterprise resilience as a dynamic capability of an enterprise which is extremely dependent on the enterprise’s employees and teams. When confronted with changes in the external environment, enterprises can develop flexible and innovative solutions to address the implications of these changes. Although these viewpoints are that resilience is regarded as a framework or capacity of enterprises, they lack effective tools to measure it. In addition, Lengnick-Hall et al. (2011) considers that the concept of enterprise resilience has two aspects. One is that it is the ability of an enterprise to recover from the unexpected events, which focuses on the response strategies and recovery speed. And another is that enterprise resilience refers to the ability to take advantage of unexpected challenges and changes to revolutionize the enterprise to reach a new state, the emphasis of which is not only on restoration but also on exploitation and expansion capabilities. Therefore, resilience is seen as an important factor for the enterprise to take advantage of uncertain opportunities and resources to overcome current challenges and achieve a higher level of enterprise status. This view is adopted in this paper, but at the same time, Lengnick-Hall et al. (2011) connects human resources systems to organizational resilience, which is considered that it is lack of actual verification in enterprise operation. Furthermore, Akgün (2014) considers enterprise resilience to be the ability to effectively absorb the negative effects of disruptive events, to react to emergencies, and, ultimately, to carry out optimal innovation through the process of dealing with unexpected events. Multilevel regression analysis is used to study the relationships between firm elasticity, product innovation, and firm performance in his paper. However, the internal structures between these variables are not investigated. Kim (2021) believed that enterprise resilience can be built through the strategic internal communication and organization–employee relationships.

Meanwhile, HRM practices are the general term of various policies, means and systems that influencing employees’ attitudes, behaviors and performance, which affect enterprise performance by impacting the employee turnover rate and enterprise productivity (Huselid, 1995). Some researchers believe that HRM practices may be the main sources of corporate performance growth and competitive advantage (Singh and Negi, 2013). The factors such as recruitment and selection, training, rewards and benefits performance appraisal, internal career opportunities and social exchange as a mediating variable of specific set of HRM practices in a Middle Eastern emerging market are discussed (Mohammad et al., 2020). HRM practices have been classified into transaction-based and commitment-based practices. Commitment-based HRM practices emphasize mutual employee–employer relationships focused on long-term exchange, while transaction-based HRM practices emphasize individual short-term exchange employee–employer relationships. Recent reviews of the field of HRM suggest that commitment-based practices are more likely to lead to higher firm performance, as these practices create an environment conducive to higher productivity; in contrast, transactional-based practices are perceived to limit the potential of HRM as a driving force behind firm performance (Pavlov et al., 2017). HRM practices are potentially a primary source of growth in enterprise performance and competitive advantage for enterprises (Singh and Negi, 2013). Although previous studies have observed that HRM practices can affect firm performance, there are still debates on which specific HRM practices can improve firm performance and which combinations of practices have an impact on performance (Huselid, 1995). According to the above literature, enterprise resilience and HRM practices are combined to discuss their impact on enterprise performance in this paper.

In addition, different from traditional quantitative statistical analysis methods based on linear causality, fsQCA is based on set theory analysis and Boolean algebra to construct the causality of the research problem based on small sample data. Compared to the regression analyses used in previous studies (Huselid, 1995), fsQCA has the advantage of being suitable for analyzing the probability of relationships and combinatorial effects between different causal conditions. This advantage is not obvious when analyzing the individual effects of single variables, but it is especially prominent only when analyzing the relationships between combinations of variables (Kraus et al., 2017). This approach is capable of fully exploring the causal structure between variables and investigating the combinatorial role of enterprise resilience and HRM practices in enterprise performance, so as to specifically find a variety of combinatorial configurations and alternative paths of high enterprise performance to enhance the breadth and specificity of the research results.

In the specific operation process, fsQCA divides the causal relationship between conditional variables and outcome variables into core presence, peripheral presence, core negation, and peripheral negation. The core presence indicates a strong causal relationship with the outcome, and peripheral presence indicates a weaker relationship. Meanwhile, the core negation indicates that the absence of condition variables has a strong causal relationship with the outcome variables, and the peripheral negation indicates that the absence of condition variables has a weak causal relationship with the outcome variables.

## 3. Enterprise resilience, HRM practices, and enterprise performance

**FIGURE 1:**
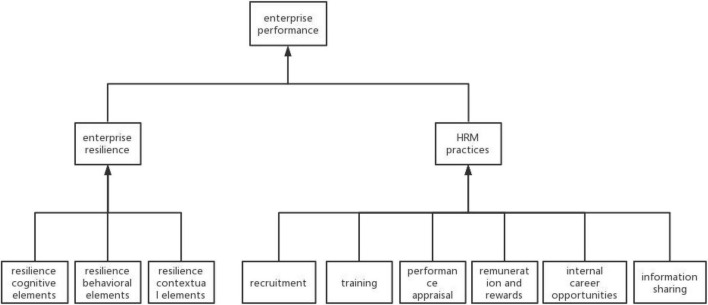
The combined effect of enterprise resilience and human resource management (HRM) practices on enterprise performance.

Research shows that both enterprise resilience and HRM practices can have a positive impact on enterprise performance (Akgün, 2014; Prayag et al., 2018). Investing in resilience capabilities can help enterprises proactively respond to the challenges of uncertainty caused by disruptive and unexpected events, improve the resource allocation capabilities and productivity within enterprises, and thus improve enterprise performance (Ates and Bititci, 2011). At the same time, investment in HRM practices can generate substantial financial returns (Katou and Budhwar, 2010). In consequence, this paper firstly analyzes the factors contained in enterprise resilience and HRM practices separately and then integrates the two. The emphasis is on the impact of the combination of these factors on enterprise performance. The theoretical model of this paper is shown in Figure 1. In the research model, Enterprise resilience capabilities consist of resilience cognitive capabilities, resilience behavioral capabilities, and resilience contextual capabilities (Lengnick-Hall et al., 2011). Based on these literatures (Huselid, 1995; Battistelli et al., 2019; Della Torre, 2019; Mohammad et al., 2020; Tensay and Singh, 2020), we analyze HRM practices from six factors, which are recruitment, training, performance appraisal, remuneration and rewards, internal career opportunities, and information sharing.

### 3.1. Enterprise resilience and enterprise performance

In an unpredictable environment, only a nimble and agile corporation can thrive and continue to develop. Enterprise resilience guides analysis and decision-making, enabling enterprises to respond to the threats and negative impacts of unexpected events. Investing in resilience helps enterprises establish competitive advantages facing the complex and volatile environment, and it improves their ability to create new opportunities.

Among the various factors of enterprise resilience, Resilience cognitive capabilities are a kind of ability of enterprise that takes core values, mission and vision as its action guidance to enable it to gain insight into unexpected events, accurately judge the current environment, and adjust resource allocation as rapidly as possible. Resilience behavioral capabilities are the daily habits of an enterprise and the inventive learning capability of its employees, enabling the enterprise to effectively respond to unexpected events, turning competitive potential into advantage and contributing to management decision-making. In addition, Resilience contextual capabilities are the ability of an enterprise’s workplace environment to positively impact its employees’ interpersonal relationships, while including the degree of separation of authority and responsibility in the enterprise. Furthermore, Resilience contextual capabilities depend on the internal and external relationships of the enterprise, which is the basis to respond effectively to unprecedentedly complex environment.

Sabatino (2016) considered that resilience can help enterprise develop appropriate strategies to address various external risks and challenges, thereby generating positive performance levels.

Based on the impact of enterprise resilience on enterprise performance, in this section, proposition P1 is proposed to develop combinatorial configurations of resilience factors that have a positive impact on enterprise performance.

r1: Enterprise can achieve high performance, while it’s in the situation of resilience contextual capabilities (peripheral negation) and resilience cognitive capabilities (core presence).
r2: Enterprise can achieve high performance, while it’s in the situation of resilience contextual capabilities (core presence) and resilience behavioral capabilities (peripheral presence).

### 3.2. HRM practices and enterprise performance

The impact of HRM practices on enterprise performance is a key topic in the field of HRM (Kleiner, 1990). And the various combinations of internal factors about HRM practices play a more prominent role in enterprise performance than a single factor (Ichniowski and Shaw, 1999). Therefore the influence of the combination of internal factors in HRM practices on enterprise performance will be discussed in the section.

Among the six factors of HRM practices, the recruitment and selection processes have a direct impact on organizational performance. An enterprise can create an effective culture through the utilization of appropriate recruitment and selection policies. The ability of a given enterprise to hire the most qualified personnel will have an effect on the attitudes of employees, interaction between the clients and the workers, and the relationships between the staff members themselves (Mohammad et al., 2020). Good training minimizes employee turnover and has a positive impact on the level of service quality in an enterprise. Good compensation and incentives may enhance the overall performance within institutions (Singh et al., 2016). Through a performance appraisal, workers in the enterprise will be able to determine the elements in their performance that will guarantee an award (Nelson and Quick, 2013). A career development pro-grammar provides an enterprise with a sustainable solution for shutting the experience and supply gap as they get ready for the future (Mohammad et al., 2021). Information sharing refers to whether an enterprise can establish an effective mechanism to enable management to clearly convey corporate strategy and performance information to employees. An efficient information sharing can make employees understand their roles in the enterprise deeply, give positive significance to their work, and promote their enthusiasm for work (Battistelli et al., 2019).

The objectives of this section are to identify the impact of different combinations of HRM practices on enterprise performance. Based on the viewpoint above, the paper proposes proposition P2: The following combinatorial configurations of HRM practices have a positive effect on enterprise performance.

t1: Enterprise can achieve high performance, while it’s in the situation of performance appraisal (core presence), training (core presence), recruitment (peripheral presence), internal career opportunities (peripheral presence), and performance appraisal (peripheral negation).
t2: Enterprise can achieve high performance, while it’s in the situation of performance appraisal (core presence), training (core presence), information sharing (peripheral presence), internal career opportunities (peripheral presence), and remuneration and rewards (peripheral presence).
t3: Enterprise can achieve high performance, while it’s in the situation of performance appraisal (core presence), training (core presence), information sharing (peripheral presence), remuneration and rewards (peripheral presence), and recruitment (peripheral presence).

### 3.3. The impact of the combination of enterprise resilience and HRM practices on enterprise performance

Based on the discussion above, this section explores the influences of the various combinations of internal factors about enterprise resilience and HRM practices on enterprise performance. Proposition P3 and proposition P4 are proposed as follows. Proposition P3:

s1: Enterprise can achieve high performance, while it’s in the situation of resilience cognitive capabilities (peripheral negation), performance appraisal (peripheral negation), information sharing (core presence), internal career opportunities (core presence), recruitment (core presence), resilience contextual capabilities (peripheral presence), resilience behavior capabilities (peripheral presence), and remuneration and rewards (peripheral presence).
s2: Enterprise can achieve high performance, while it’s in the situation of resilience contextual capabilities (peripheral negation), resilience behavior capabilities (peripheral negation), information sharing (core presence), performance appraisal (core presence), resilience cognitive capabilities (peripheral presence), remuneration and rewards (peripheral presence), training (peripheral presence), and recruitment (peripheral presence).
s3: Enterprise can achieve high performance, while it’s in the situation of remuneration and rewards (peripheral negation), internal career opportunities (core presence), performance appraisal (core presence), recruitment (core presence), resilience behavior capabilities (core presence), resilience contextual capabilities (core presence), and training (core presence).
s4: Enterprise can achieve high performance, while it’s in the situation of information sharing (core presence), internal career opportunity (core presence), performance appraisal (core presence), recruitment (core presence), remuneration and rewards (peripheral presence), training (peripheral presence), and resilience contextual capabilities (peripheral negation).
s5: Enterprise can achieve high performance, while it’s in the situation of information sharing (core presence), performance appraisal (core presence), resilience cognitive capabilities (peripheral presence), resilience behavior capabilities (peripheral presence), resilience contextual capabilities (peripheral presence), internal career opportunities (peripheral presence), remuneration and rewards (peripheral presence), and training (peripheral presence).
s6: Enterprise can achieve high performance, while it’s in the situation of information sharing (core presence), performance appraisal (core presence), resilience cognitive capabilities (peripheral presence), resilience behavior capabilities (peripheral presence), resilience contextual capabilities (peripheral presence), remuneration and rewards (peripheral presence), training (peripheral presence), recruitment (peripheral presence).

Proposition P4:

s7: Enterprise can achieve high performance, while it’s in the situation of information sharing (peripheral negation), resilience contextual capabilities (peripheral negation), resilience cognitive capabilities (peripheral negation), remuneration and rewards (peripheral negation), and training (peripheral negation).
s8: Enterprise can achieve high performance, while it’s in the situation of resilience cognitive capabilities (peripheral negation), resilience behavior capabilities (peripheral negation), resilience contextual capabilities (peripheral negation), internal career opportunities (peripheral negation), performance appraisal (peripheral negation), training (peripheral negation), recruitment (peripheral negation), information sharing (peripheral negation), remuneration and rewards (peripheral negation).

## 4. Methodology

### 4.1. Data collection

**FIGURE 2:**
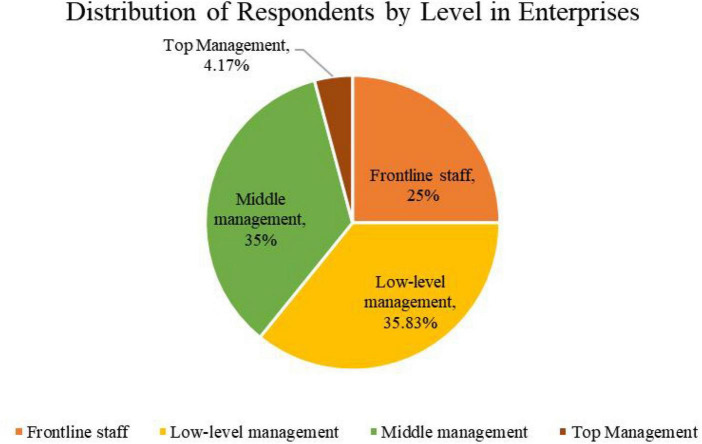
Distribution of respondents by level in enterprises.

**FIGURE 3:**
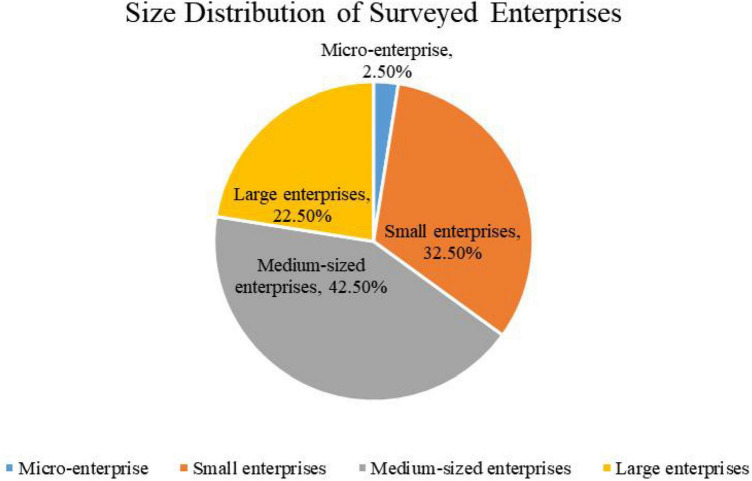
Size distribution of surveyed enterprises.

**FIGURE 4:**
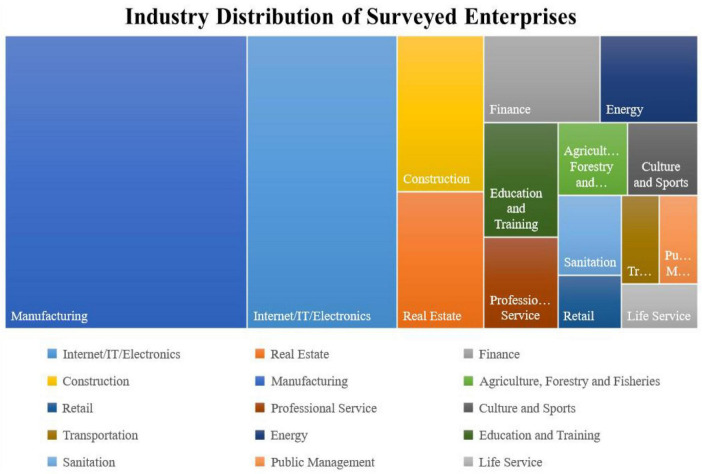
Industry distribution of surveyed enterprises.

Based on the literature, this research emphasizes covering as many industries as possible and designing a questionnaire with managers and general employees at different levels in enterprises as respondents. The survey was conducted between January and March 2021. A total of 150 questionnaires were returned, of which 119 were valid. There are 82 males and 37 females in the respondents. The distribution of the respondents by level in enterprises is shown in Figure 2. Among those who completed the questionnaire, 4% were senior managers, 35% were middle managers, 25% were low-level managers, and 25% were frontline staffs. Figure 3 shows the size of the surveyed enterprises, with 2.5% of them being micro-enterprises (The number of employees is less than 20), 32.5% being small enterprises (between 21 and 300 people), 42.5% being medium-sized enterprises (between 301 and 1,000 people), and 22.5% being large enterprises (The number of employees is more than 1,000), Figure 4 shows the industry distribution of the participating enterprises, which are located in a wide range of industries. Manufacturing enterprises account for the highest percentage (35%) of participating enterprises, 35%, followed by internet enterprises (21.67%).

### 4.2. Research method

Because Mohammad’s four-point scale contains only quantifiable performance factors, which can avoid potential common method variance, this paper uses the same method to measure the variables of HRM practices in enterprises, including six factors: recruitment, training, remuneration and rewards, performance appraisal, internal career opportunities, and information sharing. Enterprise performance is evaluated using Mohammad’s four-item scale, as shown in Supplementary Appendix A (Mohammad et al., 2020). In this paper, we adopt the variables for measuring enterprise resilience from Akgün’s (2014) study which contains 23 items to estimate the cognitive, behavioral, and contextual capabilities of enterprise resilience, as shown in Supplementary Appendix B. The respondents were asked to measure enterprise resilience, HRM practices, and enterprise performance on a 7-point Likert scale (1 = completely disagree, 7 = completely agree) based on the extent to which their enterprise conforms to the relevant issues.

### 4.3. The reliability and validity analyses

In this paper, the reliability and validity analyses of the scales were conducted using SPSS 25. Reliability analysis of the variables showed that the Cronbach alpha values for all variables were greater than 0.7 (see Table 1), ensuring the reliability of the scale. Through confirmatory factor analysis, the KMO statistic is 0.753, and the probability value of Bartlett sphericity test is 0, which is less than the significance level (0.05), indicating that there is a correlation between the variables analyzed. In total, seven factors with square roots greater than one were identified, explaining 73.4% of the variance. Thus, this scale has excellent validity.

**TABLE 1:** Reliability analysis.

Variable	α
Recruitment	0.701
Training	0.797
Performance appraisal	0.9
Remuneration and rewards	0.836
Internal career opportunities	0.917
Information sharing	0.818
Resilience cognitive capabilities	0.937
Resilience behavioral capabilities	0.937
Resilience contextual capabilities	0.949
Enterprise performance	0.926

### 4.4. fsQCA-based data analysis

fsQCA is a research method that combines qualitative and quantitative analysis. It conceptualizes *priori* cases as sets, which are used to study the combination configuration of multiple condition variables that produce the same results. In this paper, fsQCA can evaluate the results of combining three internal influencing factors of enterprise resilience with six internal influencing factors of HRM practices to produce multiple alternative paths for achieving high/non-high enterprise performance.

#### 4.4.1. Necessary and sufficient conditions

Based on fsQCA 2.5 software, first, each conditional variable is tested to determine whether it is a necessary and sufficient condition for the outcome variable (high/non-high enterprise performance). As shown in Table 2, all variables do not constitute necessary and sufficient conditions for high enterprise performance. Second, in terms of necessity, the consistency rate of all variables does not exceed the necessity threshold of 0.9. Thus, all variables in this study are not necessary conditions for high enterprise performance. From a sufficiency perspective, all individual *a priori* variables do not constitute sufficient conditions for high enterprise performance.

**TABLE 2:** Analysis of the necessary conditions for high/non-high enterprise performance.

Attribute	Outcome: jx		Outcome: ∼jx	
	Consistency	Coverage	Consistency	Coverage
RE	0.891060	0.911860	0.863556	0.406221
∼RE	0.414280	0.879097	0.816895	0.771673
TR	0.898077	0.921395	0.817117	0.386563
∼TR	0.398363	0.840869	0.835692	0.780559
PA	0.883183	0.959009	0.788501	0.391206
∼PA	0.433773	0.822298	0.911019	0.779402
RR	0.895745	0.938282	0.837240	0.398392
∼RR	0.413584	0.837587	0.853959	0.788597
ICO	0.897136	0.957630	0.777179	0.379572
∼ICO	0.410552	0.802462	0.902247	0.793588
IS	0.884083	0.956189	0.756745	0.375763
∼IS	0.418494	0.798875	0.915082	0.781750
RCG	0.886829	0.953510	0.801814	0.392592
∼RCG	0.426162	0.822107	0.892194	0.781205
RBH	0.892114	0.958060	0.798553	0.392973
∼RBH	0.428377	0.828138	0.911447	0.790944
RCT	0.894947	0.956461	0.819343	0.397480
∼RCT	0.426937	0.832626	0.892968	0.795101

RE, recruitment; TR, training; PA, performance appraisal; RR, remuneration and rewards; ICO, internal career opportunities; IS. information sharing; RCG, resilience cognitive capabilities; RBH, resilience behavioral capabilities; RCT, resilience contextual capabilities. ∼ = absence (negative).

**TABLE 3:** Configurations of enterprise resilience capabilities for high-performing enterprises.

Configuration	High performance	
	r1	r2
RCT	⊗	⚫
RBH		⬤
RCG	⚫	
Overall solution coverage: 0.87		
Overall solution consistency: 0.97		

Large circles indicate core conditions and small circles peripheral conditions. Black circles (“⬤”) indicate the “presence” of a condition, crossed-out circles (“⊗”) indicate its “negation,” and blank spaces in the solutions indicate “don’t care.”

**TABLE 4:** Configurations of human resource management (HRM) practices for high-performing enterprises.

Configuration	High performance		
	t1	t2	t3
IS		⬤	⬤
ICO	⬤	⬤	
RR	⊗	⬤	⬤
PA	⚫	⚫	⚫
TR	⚫	⚫	⚫
RE	⬤		⬤
Overall solution coverage: 0.78			
Overall solution consistency: 0.99			

Large circles indicate core conditions and small circles peripheral conditions. Black circles (“⬤”) indicate the “presence” of a condition, crossed-out circles (“⊗”) indicate its “negation,” and blank spaces in the solutions indicate “don’t care.”

**TABLE 5:** Configurations of enterprise resilience capabilities and human resource management (HRM) practices for high-performing enterprises and non-high performing enterprises.

Configuration	High Performance						Non-high Performance	
	s1	s2	s3	s4	s5	s6	s7	s8
RCT	⬤	⊗	⬤	⊗	⬤	⬤	⊗	⬤
RBH	⬤	⊗	⬤	⬤	⬤	⬤		⬤
RCG	⊗	⬤	⬤		⬤	⬤	⊗	⬤
IS	⚫	⚫		⚫	⚫	⚫	⭙	⭙
ICO	⚫		⚫	⚫	⬤			⬤
RR	⬤	⬤	⊗	⬤	⬤	⬤	⊗	⊗
PA	⊗	⚫	⚫	⚫	⚫	⚫		⬤
TR		⬤	⬤	⬤	⬤	⬤	⊗	⬤
RE	⚫	⬤	⚫	⚫		⬤		⬤
Solution coverage: 0.75							Solution coverage: 0.75	
Solution consistency: 0.99							solution consistency: 0.90	

Large circles indicate core conditions and small circles peripheral conditions. Black circles (“⬤”) indicate the “presence” of a condition, crossed-out circles (“⊗”) indicate its “negation,” and blank spaces in the solutions indicate “don’t care.”

Table 2 also shows the results of testing each conditional variable to determine whether it is a necessary and sufficient condition for non-high enterprise performance. With respect to non-high enterprise performance, except for ∼performance appraisal, ∼internal career opportunities, ∼information sharing, and ∼resilience behavioral capabilities, none of the variables exceeds the necessity threshold of 0.9 for the level of the consistency rate, and they do not constitute a necessary condition. In addition, ∼performance appraisal, ∼internal career opportunities, ∼information sharing, and ∼resilience behavioral capabilities constitute necessary conditions for non-high enterprise performance. Therefore, to a significant extent, the absence of performance appraisal, internal career opportunities, information sharing, and resilience behavioral capabilities results in non-high enterprise performance. When performing fsQCA, we need to incorporate these conditions.

#### 4.4.2. Setting qualitative anchors

In this paper, a direct calibration method is used to identify the affiliation degree. This procedure requires specifying the value of an interval scale variable that corresponds to the three qualitative anchors that constitute the fuzzy set (ragin 2008), which are full membership in the set (fuzzy score = 0.95), full non-membership in the set (fuzzy score = 0.05), and intersection (fuzzy score = 0.5). The measurement scale is converted into a fuzzy set affiliation score when measuring the level of HRM practices, enterprise resilience, and enterprise performance *via* a 7-point Likert scale. Full membership with a fuzzy affiliation score of 0.95 is set to 7 (fully agree); the crossover point for membership with a fuzzy affiliation score of 0.5 is set to 4 (between agree and disagree); full non-membership with a fuzzy affiliation score of 0.05 is set to 1 (completely disagree). For example, when the respondents rate recruitment as 7 (fully conforming), this rating indicates that the respondents believe that the recruitment aspect of their enterprise HRM practices is performing at a very high level.

## 5. Results and discussion

The results were obtained using fsQCA (fsQCA 2.5) and are shown in Tables 3–5. Table 3 shows the resilience capability configurations of high-performing enterprises, Table 4 shows the HRM practices configurations of high-performing enterprises, and Table 5 shows the resilience and HRM practices configurations of high-/non-high-performing enterprises. Besides, Table 3 shows the impact of enterprise resilience capabilities on high enterprise performance. For r1, resilience contextual capabilities were absent as a peripheral condition, and resilience cognitive capabilities were present as a core condition. For r2, resilience contextual capabilities were present as a core condition, and resilience behavioral capabilities were present as a peripheral condition. Both r1 and r2 are resilience capability configurations for high-performing enterprises. The impact of HRM practices on high enterprise performance obtained results t1, t2, and t3, as shown in Table 4. Performance appraisal and training are present as core conditions in t1, t2, and t3. Thus, it is concluded that performance appraisal and training have a significant positive effect on high enterprise performance.

In this study, the consistency threshold was set to 0.8 in fsQCA (Ragin, 2008) and the PRI threshold was set to 0.8, while the frequency of acceptable cases was set to 1. The results obtained are shown in Table 5. The results of the analysis found a total of six paths for achieving high enterprise performance: s1, s2, s3, s4, s5, and s6. The consistency values for each path and the overall solution exceeded 0.8, and these six configurations accounted for 75% of the overall membership. These results suggest that these six configurations are capable of achieving high levels of enterprise performance. Furthermore, as shown by the results in Table 5, the overall consistency is 0.90, which is higher than the consistency threshold of 0.8, and the coverage reaches 0.77. Two configurations, s7 and s8, for achieving non-high enterprise performance are obtained. Thus, the correctness of propositions P1-P4 is verified.

Based on these results, it is concluded that information sharing capabilities play a critical role in achieving high enterprise performance. Information sharing capabilities are present as a core condition in five of the six configurations that achieve high enterprise performance, while it is also absent as a core condition in all two configurations that achieve non-high enterprise performance. This finding indicates that information sharing capabilities have a significant positive impact on enterprise performance, in addition, in the six configurations for achieving high enterprise performance, resilience cognitive capabilities, resilience behavioral capabilities, and resilience contextual capabilities all exist as auxiliary conditions in three cases, configurations s3, s5, and s6, indicating that enterprise resilience capabilities have a relatively positive impact on enterprise performance.

Moreover, through the group regression analysis and regression difference test of data at different levels, it can be concluded that middle managers, low-level managers and frontline staffs have the same views about the impact of various factors on enterprise performance, as shown in Supplementary Appendix C and Table 6.

**TABLE 6:** Results of group regression and difference test at different levels.

	(1)	(2)	(3)
	Enterprise Performance	Enterprise Performance	Enterprise Performance
Information Sharing	0.206 (0.97)	−0.223 (−1.02)	−0.217 (−1.04)
Internal career opportunities	0.399 (1.65)	0.231 (1.14)	0.400* (1.99)
Remuneration and rewards	0.119 (0.58)	0.0848 (0.56)	0.314 (1.64)
Performance appraisal	−0.122 (−0.89)	0.00437 (0.03)	−0.00129 (−0.01)
Training	–0.121 (−0.67)	0.170 (0.99)	0.138 (0.87)
Recruitment	0.543*** (3.63)	0.246 (1.40)	0.325** (2.12)
Context	0.521*** (3.14)	0.456** (2.67)	0.212 1.43)
Behavior	−0.205 (−0.70)	−0.0190 (−0.14)	−0.0627 (−0.47)
Cognition	0.209 (1.12)	−0.0347 (−0.24)	−0.185 (−1.41)
_cons	−2.854 (−1.22)	0.440 (0.18)	0.273 (0.11)
*N* r2	26 0.701	35 0.326	34 0.414

*t* statistics in parentheses, *p < 0.1, ^**^p < 0.05, ^***^p < 0.01.

In this paper, the proof of propositions t3, s3 and s4 verifies Mohammad et al.’s (2020) research that recruitment, training, and internal career opportunities can play a positive and significant role in enterprise performance, and on the basis of which, the influence of enterprise resilience on enterprise performance is strengthened. For example, resilience cognitive capabilities, resilience behavior capabilities, and resilience contextual capabilities are all peripheral presence in proposition s3, while resilience contextual capabilities are core negation and resilience behavior capabilities are peripheral presence in s4. However, both s3 and s4 can achieve high enterprise performance. This means we conclude that the paths to high enterprise performance are not only affected by factors of enterprise resilience or factors of HRM practices but also by combining the internal factors of HRM practices with enterprise resilience. As a result, it is more diverse than before for the paths to high enterprise performance. Therefore, managers need to seek the development of enterprise resilience and HRM practices simultaneously, and choose the most suitable combination configuration according to the actual situation of the enterprise itself.

In addition, it can be seen from the research results of this paper that information sharing keeps a core presence in the five high enterprise performance paths, indicating that enterprises need to establish an effective information-sharing mechanism to obtain high enterprise performance. For this reason, the suggestion is made to set up a meeting system aimed at conveying corporate strategy and performance information from management to general employees and feedback from general employees to management regularly, while establishing a complete meeting minutes and review mechanism to ensure the transmission of internal information efficiently and accurately.

## 6. Research significance

The research above shows that the path to high enterprise performance is complex and multifaceted. Neither enterprise resilience nor HRM practices by themselves are sufficient conditions for high-level enterprise performance. The same result can be obtained with different configurations of enterprise resilience capabilities and HRM practices. The same results (high/non-high enterprise performance) are achieved by different combinations of enterprise elasticity and internal factors of HRM practices, which can certify the positive combined effects of enterprise resilience and HRM practices on enterprise performance.

In this paper, the influence of enterprise resilience and HRM practices on enterprise performance is studied. By using fsQCA method, different paths to achieve high/non-high enterprise performance are analyzed. And the causal relationship between variables is analyzed from the inside of enterprises, which provides valuable reference for the development of enterprise resilience and investment in HRM practices. In the future, time series data can be used to compare with the results of this paper and it is possible to choose different combination configurations about enterprise resilience and HRM practices to study enterprise performance in different backgrounds.

## Data availability statement

The raw data supporting the conclusions of this article will be made available by the authors, without undue reservation.

## Author contributions

LT and BL proposed the idea, collected the data, and preformed the statistical analysis. YL and LT drafted the manuscript and designed the questionnaires. HC and YZ revised the manuscript. BL and YL designed the study, contributed to the revision of the manuscript, and funded the investigation. All authors have read and agreed to the final version of the manuscript.
