# Supplementary material for: The impact of enterprise resilience and HRM practices on performance: Findings from fsQCA
Source: Front Psychol. 2023 Feb 13;14:1114087. doi: 10.3389/fpsyg.2023.1114087 (PMC9968964; doi:10.3389/fpsyg.2023.1114087)
Supplement: Supplementary file 1 [file Data_Sheet_1.DOC]

**Appendix A**

Appendix B

| Human resource management practices and enterprise performance measurement scale | |
| --- | --- |
| Constructs | Items |
| Recruitment |  |
|  | When filling vacant positions, the enterprise would like to promote from within |
|  | …prefers to recruit people with creative thinking skills |
|  | …prefers to recruit employees with specialized skills |
|  | The recruitment process will adapt the candidate to the job |
| Training |  |
|  | The enterprise provides continuous training to update the skills and knowledge of its employees |
|  | The enterprise provides equal opportunities for all employees |
|  | Training sessions familiarize new employees with organizational norms and values |
|  | All training programs are of high quality |
| Performance appraisal |  |
|  | The standards in employee evaluation are clear |
|  | Performance appraisals can have a positive impact on employees’ commitment to work |
|  | Management provides regular feedback |
|  | Employees are satisfied with the results of their performance appraisals |
|  | Performance appraisals are results oriented |
| Remuneration and rewards |  |
|  | Good work performance is noticed and rewarded |
|  | Top management prefers to be involved in decision-making at all levels of work |
|  | Work performance is an important factor in determining employee rewards and remuneration |
|  | The remuneration system provides rewards for innovative ideas |
|  | Corporate welfare systems related to knowledge and skill levels provide rewards for the knowledge and skills of employees |
| Internal career opportunities |  |
|  | Qualified employees have the opportunity to advance to a higher salary and/or responsible positions within the organization |
|  | There are multiple potential positions for promotion for employees who wish to advance in this enterprise |
|  | Training in the field of work is well supported |
|  | Our enterprise provides support to keep up with the newest developments in the industry |
|  | Our enterprise's promotion process is fair to all employees |
| Information sharing |  |
|  | Management can clearly and accurately communicate the organization's competitive strategy to employees |
|  | Management can clearly and accurately communicate to employees their performance information |
| Enterprise performance |  |
|  | The quality of products and services |
|  | Profitability |
|  | Market share |
|  | Sales growth |
|  | Employee productivity |

| Enterprise resilience scale | |
| --- | --- |
| Constructs | Items |
|  |  |
| Resilience cognitive capabilities |  |
|  | We have a strong sense of purpose, clear core values and a defined vision in our organization. |
|  | In times of difficulty, the core values, mission and sense of recognition given to us by the organization encourage us to take positive action to solve problems. |
|  | In the enterprise, there is a buzzword that represents the impact, core values and direction of the enterprise. |
|  | We have a very clear moral purpose that allows us to motivate our employees and enable them to make full use of their mental and physical resources. |
|  | We have the mindset to question problems in the enterprise. |
|  | In the enterprise, we encourage innovation and the development of new technologies rather than emphasizing the necessity of control and standardization. |
|  | When problems arise in the enterprise, we focus on a specific analysis of specific issues rather than procedural solutions. |
|  | In the enterprise, we use a common language (words, images and stories) to construct meaning, describe scenarios and communicate emotions. |
| Resilience behavioral capabilities |  |
|  | When faced with unprecedented challenges, employees are able to develop unconventional and powerful responses through disciplined creativity. |
|  | When faced with difficulties, employees are able to combine initiative with originality to take advantage of the current conditions in our organization. |
|  | We have the ability to follow a dramatically different course of action from that which is the norm in our organization. |
|  | We have a complex and varied action inventory that enables us to follow a dramatically different course of action from that which is the norm in our organization. |
|  | We have a number and diversity of competitive actions available to adopt unexpected and timely responses to market shifts in our organization. |
|  | We develop values that lead to habits of investigation rather than assumptions in our organization. |
|  | We develop values that lead to routines of collaboration rather than antagonism in our organization. |
|  | We develop values that lead to traditions of flexibility rather than rigidity in our organization. |
|  | We take actions and make investments before they are needed to ensure that we are able to benefit from situations that emerge in our organization. |
|  | We quickly discard behaviors that constrain us because it is for us to develop new competencies in our organization. |
|  | In our organization, we have the ability to spot an opportunity that other firms without our competencies might miss. |
| Resilience contextual capabilities |  |
|  | People perceive their work environment as being conducive to taking interpersonal risks, such as the risk of being seen as ignorant by asking questions or seeking information, in our organization. |
|  | People perceive their work environment as being conducive to taking interpersonal risks, such as the risk of being seen as incompetent as a result of asking for help, admitting mistakes or experimenting, in our organization. |
|  | People perceive their work environment as being conducive to taking interpersonal risks, such as the risk of being seen as negative when offering critical feedback, in our organization. |
|  | Employees have mutually respectful interactions in the organization. |
|  | There is an ongoing conversation among employees based on trust, honesty and self-esteem. |
|  | Employees exchange resources and share information with each other. |
|  | There is cross-functional collaboration in the organization. |
|  | Each member of the organization has both the discretion and the responsibility to ensure attainment of organizational interests in our organization. |
|  | We create organizational structures that are designed to learn and to change employee behaviors based on new insights and information. |
|  | We share decision-making widely. |
|  | People forge relationships with others who can share key resources. |
|  | We use relationships with supplier contacts and strategic alliances to secure needed resources to support adaptive initiatives. |

**Appendix C**

| . test [t1_mean] information sharing=[t2_mean] information sharing |
| --- |
| ( 1) [t1_mean] information sharing - [t2_mean] information sharing = 0 |
| chi2( 1) = 3.08 |
| Prob > chi2 = 0.0792 |
| . test [t1_mean] internal career opportunities=[t2_mean] internal career opportunities |
| ( 1) [t1_mean] internal career opportunities - [t2_mean] internal career opportunities = 0 |
| chi2( 1) = 0.49 |
| Prob > chi2 = 0.4856 |
| . test [t1_mean] remuneration and rewards =[t2_mean] remuneration and rewards |
| ( 1) [t1_mean] remuneration and rewards - [t2_mean] remuneration and rewards = 0 |
| chi2( 1) = 0.03 |
| Prob > chi2 = 0.8621 |
| . test [t1_mean] performance appraisal=[t2_mean] performance appraisal |
| ( 1) [t1_mean] performance appraisal - [t2_mean] performance appraisal = 0 |
| chi2( 1) = 0.45 |
| Prob > chi2 = 0.5021 |
| . test [t1_mean] training=[t2_mean] training |
| ( 1) [t1_mean] training - [t2_mean] training = 0 |
| chi2( 1) = 1.89 |
| Prob > chi2 = 0.1696 |
| . test [t1_mean] recruitment=[t2_mean] recruitment |
| ( 1) [t1_mean] recruitment - [t2_mean] recruitment = 0 |
| chi2( 1) = 2.23 |
| Prob > chi2 = 0.1355 |
| . test [t1_mean] context=[t2_mean] context |
| ( 1) [t1_mean] context - [t2_mean] context = 0 |
| chi2( 1) = 0.14 |
| Prob > chi2 = 0.7086 |
| . test [t1_mean] behavior=[t2_mean] behavior |
| ( 1) [t1_mean] behavior - [t2_mean] behavior = 0 |
| chi2( 1) = 0.77 |
| Prob > chi2 = 0.3799 |
| . test [t1_mean] cognition =[t2_mean] cognition |
| ( 1) [t1_mean] cognition - [t2_mean] cognition = 0 |
| chi2( 1) = 2.51 |
| Prob > chi2 = 0.1131 |
| . test [t1_mean] information sharing =[t3_mean] information sharing |
| ( 1) [t1_mean] information sharing - [t3_mean] information sharing = 0 |
| chi2( 1) = 2.95 |
| Prob > chi2 = 0.0857 |
| . test [t1_mean] internal career opportunities =[t3_mean] internal career opportunities |
| ( 1) [t1_mean] internal career opportunities - [t3_mean] internal career opportunities = 0 |
| chi2( 1) = 0.00 |
| Prob > chi2 = 0.9953 |
| . test [t1_mean] remuneration and rewards =[t3_mean] remuneration and rewards |
| ( 1) [t1_mean] remuneration and rewards - [t3_mean] remuneration and rewards = 0 |
| chi2( 1) = 1.00 |
| Prob > chi2 = 0.3162 |
| . test [t1_mean] performance appraisal =[t3_mean] performance appraisal |
| ( 1) [t1_mean] performance appraisal - [t3_mean] performance appraisal = 0 |
| chi2( 1) = 0.46 |
| Prob > chi2 = 0.4958 |
| . test [t1_mean] training =[t3_mean] training |
| ( 1) [t1_mean] training - [t3_mean] training = 0 |
| chi2( 1) = 1.61 |
| Prob > chi2 = 0.2039 |
| . test [t1_mean] recruitment =[t3_mean] recruitment |
| ( 1) [t1_mean] recruitment - [t3_mean] recruitment = 0 |
| chi2( 1) = 1.55 |
| Prob > chi2 = 0.2130 |
|  |
| . test [t1_mean] context =[t3_mean] context |
| ( 1) [t1_mean] context - [t3_mean] context = 0 |
| chi2( 1) = 3.55 |
| Prob > chi2 = 0.0595 |
| . test [t1_mean] behavior =[t3_mean] behavior |
| ( 1) [t1_mean] behavior - [t3_mean] behavior = 0 |
| chi2( 1) = 0.51 |
| Prob > chi2 = 0.4748 |
| . test [t1_mean] cognition =[t3_mean] cognition |
| ( 1) [t1_mean] cognition - [t3_mean] cognition = 0 |
| chi2( 1) = 6.41 |
| Prob > chi2 = 0.0114 |
| . test [t3_mean] information sharing =[t2_mean] information sharing |
| ( 1) [t3_mean] information sharing - [t2_mean] information sharing = 0 |
| chi2( 1) = 0.00 |
| Prob > chi2 = 0.9826 |
| . test [t3_mean] internal career opportunities =[t2_mean] internal career opportunities |
| ( 1) [t3_mean] internal career opportunities - [t2_mean] internal career opportunities = 0 |
| chi2( 1) = 0.49 |
| Prob > chi2 = 0.4860 |
| . test [t3_mean] remuneration and rewards =[t2_mean] remuneration and rewards |
| ( 1) [t3_mean] remuneration and rewards - [t2_mean] remuneration and rewards = 0 |
| chi2( 1) = 1.59 |
| Prob > chi2 = 0.2075 |
| . test [t3_mean] performance appraisal =[t2_mean] performance appraisal |
| ( 1) [t3_mean] performance appraisal - [t2_mean] performance appraisal = 0 |
| chi2( 1) = 0.00 |
| Prob > chi2 = 0.9796 |
| . test [t3_mean] training =[t2_mean] training |
| ( 1) [t3_mean] training - [t2_mean] training = 0 |
| chi2( 1) = 0.03 |
| Prob > chi2 = 0.8697 |
| . test [t3_mean] recruitment =[t2_mean] recruitment |
| ( 1) [t3_mean] recruitment - [t2_mean] recruitment = 0 |
| chi2( 1) = 0.15 |
| Prob > chi2 = 0.7010 |
| . test [t3_mean] context =[t2_mean] context |
| ( 1) [t3_mean] context - [t2_mean] context = 0 |
| chi2( 1) = 1.78 |
| Prob > chi2 = 0.1823 |
| . test [t3_mean] behavior =[t2_mean] behavior |
| ( 1) [t3_mean] behavior - [t2_mean] behavior = 0 |
| chi2( 1) = 0.09 |
| Prob > chi2 = 0.7629 |
|  |
| . test [t3_mean] cognition =[t2_mean] cognition |
| ( 1) [t3_mean] cognition - [t2_mean] cognition = 0 |
| chi2( 1) = 0.84 |
| Prob > chi2 = 0.3591 |
